# Recognizing wetland ecosystem services for sustainable rice farming in the Mekong Delta, Vietnam

**DOI:** 10.1007/s11625-016-0409-x

**Published:** 2016-11-02

**Authors:** Håkan Berg, Agnes Ekman Söderholm, Anna-Sara Söderström, Nguyen Thanh Tam

**Affiliations:** 1 Department of Physical Geography, Stockholm University, SE-106 91 Stockholm, Sweden; 2 Faculty of Fishery, Nong Lam University, Block 6, Linh Trung Ward, Thu Duc District, HCM City, Vietnam

**Keywords:** Rice farming, Rice–fish, Integrated pest management, Ecosystem services, Pesticides, Mekong Delta

## Abstract

The increased rice production in the Mekong Delta during the last two decades has improved agricultural income and reduced poverty, but it has also had negative impacts on the environment and human health. This study shows that integrated rice–fish farming and integrated pest management strategies provide sustainable options to intensive rice farming, because of a more balanced use of multiple ecosystem services that benefit the farmers’ health, economy and the environment. The study investigates and compares farming strategies among 40 rice and 20 rice–fish farmers in two locations in the Mekong Delta. Production costs and income are used to compare the systems’ financial sustainability. The farmers’ perception on how their farming practices influence on ecosystem services and their livelihoods are used as an indication of the systems’ ecological and social sustainability. Although rice–fish farmers used lower amount of pesticides and fertilisers than rice farmers, there were no statistical differences in their rice yields or net income. Rice was seen as the most important ecosystem service from rice fields and related wetlands, but also several other ecosystem services, such as water quality, aquatic animals, plants, habitats, and natural enemies to pests, were seen as important to the farmers’ livelihoods and wellbeing. All farmers perceived that there had been a general reduction in all these other ecosystem services, due to intensive rice farming during the last 15 years, and that they will continue to decline. The majority of the farmers were willing to reduce their rice yields slightly for an improved quality of the other ecosystem services.

## Introduction

Vietnam started to export rice in the late 1980s and is now one of the largest rice exporters in the world (Renaud and Kunezer 2012). The Mekong Delta is the most important region for rice production in Vietnam, and supplies some 50% of the national rice production (Sebesvari et al. 2012). Increased rice yields have been achieved through more intensive farming methods, with two or three crops per year, and increased use of pesticides and fertilisers (Berg 2002; Duong et al. 2005; Sebesvari et al. 2012; UNEP 2005). This has contributed to increased agricultural income and reduced poverty, but it has also been followed by negative impacts on the environment and people’s health (Berg and Tam 2012; Dasgupta et al. 2007), which in the long run could impact on the overall production and quality of agriculture and aquaculture products from the Delta (cf. Luo et al. 2014). Tam et al. (2015) reported that farmers spraying organophosphates on rice fields resulted in both reduced growth and survival rates of fish, and Dasgupta et al. (2007) found that over 35% of 190 rice farmers in the Mekong Delta, experienced acute pesticide poisoning, and that 21% were chronically poisoned.

To halt this trend, there is a need to develop and adopt more sustainable rice farming practices in the region, which can maintain a high production and well-functioning ecosystem services for the benefit of people’s livelihoods and wellbeing (Berg et al. 2012; Johnston et al. 2010; Luo et al. 2014; Sebesvari et al. 2012; Zheng et al. 2016). The importance of wild aquatic organisms to poor peoples’ livelihoods and ecosystem functions must, for example, be recognised when developing future rice farming strategies.

Integrated systems with rice and fish in combination with IPM strategies (Integrated Pest Management) have been suggested to provide economically, ecologically and socially sustainable alternatives to rice monoculture, since these systems require less pesticides and fertilisers, and provide a diversified income from both fish and rice, and have less negative impacts on the environment and people’s health (Berg 2002; Duong et al. 1998; Luo et al. 2014; Xie et al. 2011; Zheng et al. 2016). An increased mixture of rice and aquaculture systems could also increase the farmers’ income and their adaptability to climate change and changes in river flows, linked to up-streams dams (Smajgl et al. 2015).

In this study, we compare such integrated systems with more intensive rice monocropping strategies, to elucidate how these systems are linked to the environment, ecosystem services and people’s wellbeing. The study focuses on ecosystem services from rice fields and related wetlands in the Mekong Delta, and includes examples of provisioning, regulating, supporting and cultural services. The study shows that rice farming not only depends on ecosystem services for an efficient production of rice and associated products, such as fish, but also impacts on several ecosystem services that are of key importance to people’s livelihood and wellbeing in the Mekong Delta.

A sustainable development of rice farming must, therefore, take into account the societal value of ecosystem services for an efficient and environmentally sound production of food. Otherwise, there is a risk that short-term gains, based on intensive ecosystem exploitations, will disrupt ecological functions and, in turn, potentially create economic and social problems. A sustainable food production in the Mekong Delta should have the aim to reduce the resource use, avoid overuse of agrochemicals and improve the production efficiency through increased recycling of nutrients and matter.

The successful adoption of such systems requires that they are financially competitive to more intensive methods, and in this study, we, therefore, compare the production cost and income of intensive rice farming with more integrated farming strategies, to assess to what extent these systems can provide financially sustainable alternatives to intensive rice farming.

The ecological sustainability of these systems is assessed by comparing the farmers’ perception of how these systems impact on human health and the environment, and to what extent they enhance or degrade ecosystem services. The social sustainability of the different systems is indicated by the farmers’ choice of the different farming systems, and their potential to contribute to diversified livelihoods and increased resilience to future changes, such as upstream dams and climate change.

## Methods

### Study area

The Mekong Delta is home for 17.4 million people making up 23% of Vietnam’s population (Nguyen and Woodroffe 2015). It covers an area of four million hectares and is one of the poorest regions in Vietnam (Renaud and Kunezer 2012). The population is highly dependent on the river and its natural resources for their livelihoods and wellbeing. The central government plans have dedicated approximately 1.8 million ha of agriculture land in the Mekong Delta to rice production, with an annual target production of 23 million tons of rice for domestic consumption and export (Smajgl et al. 2015). The Delta is a flat and low-lying region with an elevation of 0–4 m above mean sea level, with a high risk to be heavily impacted by climate change and upstream dams (Kunezer and Renaud 2012; MRC 2010; Nguyen and Woodroffe 2015; Smajgl et al. 2015; Tessler et al. 2016).

**Fig. 1:**
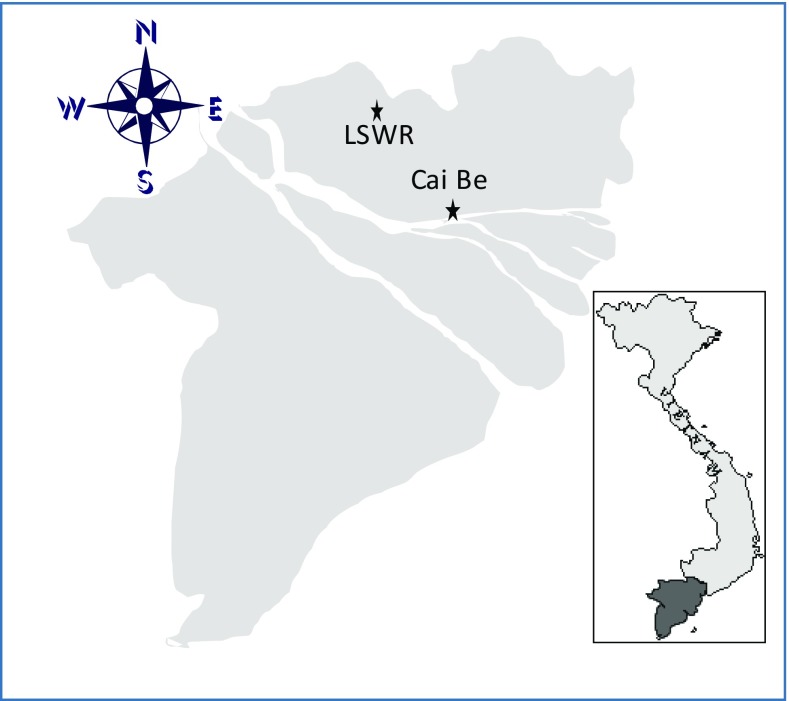
The Mekong Delta and the location of Cai Be in the Tiên Giang province and the Lang Sen Wetland Reserve (LSWR) in the Long An province, where the research was conducted

The field surveys were carried out in the Cai Be district in the Tien Giang province and the Lang Sen Wetland Reserve (LSWR) in the Long An province (Fig. 1). Tien Giang is more densely populated than Long An, but has a lower production of rice (Table 1).

**Table 1:** Some characteristics of Tiên Giang and Long An provinces (General Statistics Office of Vietnam 2014)

	Tiên Giang	Long An
Area of province (km^2^, 2011)	2508.3	4492.4
Population size (1000 people, 2011)	1682.6	1449.6
Population density (person/km^2^, 2011)	671.0	323.0
Planted area of paddy (1000 ha, 2011)	241.8	484.2
Production of paddy (1000 tons, 2011)	1332.8	2550.7
Production of aquaculture fish (1000 tons, 2011)	90,706.0	23,093.0

Cai Be is a representative rice-producing area in the Mekong Delta with both intensive rice farming and integrated rice–fish farming (Berg and Tam 2012). The area around Cai Be has a very good irrigation system consisting of a network of many canals and natural rivers.

The Lang Sen Wetland Reserve (LSWR) covers 3280 ha and is one of the few remaining natural wetlands in the area (Linstead et al. 2006). Most rice production is located in the northeast part of the area. The relatively high biodiversity found in LSWR is important for local people’s livelihoods, and fish are the most significant protein source for the people in LSWR (Nguyen and Wyatt 2006). The LSWR represents an area with a relatively short history of agricultural practices and resembles a natural wetland, and provided an opportunity to compare these farmers’ agriculture strategies and perceptions of ecosystem services with the farmers from the more intensified agricultural area of Cai Be.

### Research design

The study was conducted in 2014 and included a total of 60 rice farmers from Cai Be and Lang Sen (Table 2). The farmers were chosen according to the type of rice production system they had, and focused on monoculture rice farming and integrated rice–fish farming with and without integrated pest management strategies (IPM). The farmers were identified with the help of local extension officers from the area. All respondents were male farmers, which may have influenced on the answers related to ecosystem services.

**Table 2:** Farmers from Cai Be and Lang Sen involved in the study

Type of farmer	Cai Be	Lang Sen Wetland Reserve (LSWR)
Rice farmer (R)	20 (14 with IPM)	20 (0 with IPM)
Rice–fish farmer (RF)	20 (20 with IPM)	0

*RF* farmers cultivating rice and farming fish, *R* farmers cultivating only rice, *IPM* Integrated Pest Management

### Farming practices

Technical and financial aspects of the different farming categories were assessed through field observations, questionnaires and group discussions, and provided a basis to compare the financial sustainability of the different farming strategies.

The production costs were estimated from the input of fertilisers, pesticides, rice seeds, fish fingerlings, fish feed, water and labour. The income was estimated from the production of one crop of rice and one crop of fish. As the farmers only had one crop of fish per year, but up to three crops of rice per year, the income and cost from the fish crop were divided by three.

To get a background on the interviewed farmers groups, general information about household size, farm area and educational level was collected through questionnaires. Health aspects, particularly in relation to pesticides use, were also covered in the questionnaires. The farmers were also asked about their views on their future plans for rice farming and rice–fish farming.

### Farmers’ perception of ecosystem services

To assess the farmers’ perception of ecosystem services associated with rice fields and related wetlands, and the importance of these to their livelihoods and wellbeing, the farmers were asked to list all of the benefits that they gained from their rice fields besides rice. These benefits/ecosystem services were then compiled into a list, from which the farmers were asked to rank the identified ecosystem services in order of importance to their wellbeing and livelihoods. They were also asked to estimate how these ecosystem services had changed over time and how they thought these would change in the future. The participation of farmers assured inclusion of local knowledge in the assessment, which has been recognised as very important both within the Intergovernmental Platform on Biodiversity and Ecosystem Services (IPBES) and the Intergovernmental Platform on Climate Change (IPCC) for local assessment and scenario analysis (Kok et al. 2016; Vogt et al. 2016).

Compared to the financial analysis above, which primarily focused on the technical and financial aspects of producing rice and fish (i.e., provisioning ecosystem services), this section focused on a broader set of ecosystem services. It assessed to what extent farmers were aware of these ecosystem services; if they were seen as an integrated part of their farming strategies; and how the farmers perceived possible trade-offs in these ecosystem services under the different farming strategies. Thus, this section addressed more on the ecological and social sustainability of the different farming systems. This was also investigated in semi-structured interviews, that enabled discussions around topics that the respondent felt confident in, and gave a deeper insight into the farmers’ knowledge on specific topics (Potter and Desai 2006).

Group meetings, focusing on farming systems and ecosystem services were also held in Cai Be (3 rice–fish farmers and 10 rice farmers) and in LSWR (6 rice farmers). These farmers had previously answered the questionnaire, and were now given more in-depth follow-up questions to discuss jointly. This gave a deeper insight on their views of benefits from the rice fields and constraints to their farming activities. The group meetings lasted for 2–3 h. The group meetings also provided an opportunity to explain the concept of cultural ecosystem services, which initially was less clear to the farmers. However, after providing examples of cultural ecosystem services such as recreation, festivals and traditions linked to the landscape, the farmers could provide their own experience and examples on these services. Overall, the guided discussions seemed to only have had a minor influence on the farmers’ own perspectives on these matters.

### Farmers’ choice of future farming systems

After the questionnaires and group discussions, the farmers were asked to choose between two scenarios of future farming practices. The first scenario built on current intensive rice farming strategies, with high rice yields, but also with negative effects on the other ecosystem services, as perceived by the farmers. The second scenario built on more integrated extensive rice farming practices, such as rice–fish farming and IPM, with somewhat lower rice yield, but with improved quality of the other ecosystem services.

As the choice of the future farming systems was seen as a way for the farmers to balance financial benefits with more environmental and social benefits, the choice of the farming system was seen as an indication of the different farming systems’ overall sustainability.

### Data analysis and statistics

The results from the questionnaires, semi-structured interviews and group meetings were translated into English by Vietnamese researchers, who had participated in the consultations. All statistical analysis was made with one-way analysis of variance (ANOVA) and Dunnett’s post hoc test for multiple comparisons. SPSS for windows (Ver 17.0; SPSS, Chicago, IL, USA) was used to analyze the data.

## Results

### Yield of rice and fish

The farmers in the two districts shared many basic characteristics (Table 3). They used almost the same amount of rice seeds, and the rice yields did not differ between the groups of farmers (*P* > 0.05, Table 4). The slightly lower yields among the rice–fish farmers could be explained by the fact that part of the field was used for the fish canal.

**Table 3:** General information about rice–fish (RF) and rice (R) farmers in the Cai Be district and rice (R) farmers in the Lang Sen Wetland Reserve (LSWR)

	Type of farmer		
	Cai Be		LSWR
	RF	R	R
Household size (number of people)			
Mean	4.6	4.1	4.7
SD	1.1	1.4	1.3
Educational level (years)			
Mean	8.4	8.3	7.6
SD	2.6	2.8	3.2
Experience in rice farming (years)			
Mean	29.1^a^	20.8^b^	16.8^b^
SD	8.9	8.1	6.4
Experience in rice–fish farming (years)			
Mean	5.8	–	–
SD	4.0	–	–
Number of people involved in rice farming			
Mean	2.4	1.9	2.1
SD	1.4	0.7	0.9
Total farm area (ha)			
Mean	1.1^a^	1.3^a^	5.6^b^
SD	0.6	0.7	5.8

Means within the same row that do not share the same superscript letter are significantly different (*P* < 0.05)

*RF* farmers cultivating rice and farming fish, *R* farmers cultivating only rice

**Table 4:** Rice seeds, fish fingerlings and yield of rice and fish (kg ha^−1^ crop^−1^) among rice–fish (RF) and rice (R) farmers in Cai Be and rice (R) farmers in LSWR

	Type of farmer		
	Cai Be		LSWR
	RF	R	R
Rice seeds			
Mean	130.0	142	121
SD	12.8	38.6	23.6
Rice yields			
Mean	6806	7629	7423
SD	1221	1905	684
Fish fingerlings			
Mean	76	–	–
SD	21		
Fish yields			
Mean	966	–	–
SD	498		

Means within the same row that do not share the same superscript letter are significantly different (*P* < 0.05)

*RF* farmers cultivating rice and farming fish, *R* farmers cultivating only rice

The two most common fish species grown among the rice–fish farmers were Snakeskin gourami (*Trichopodus pectoralis*) and Climbing perch (*Anabas testudineus*), with an average yield of 586 and 142 kg per hectare, respectively. Only three of the rice–fish farmers grew other fish species, such as grass carp (*Ctenopharyngodon idella*) and common carp (*Cyprinus carpio*).

The majority (80%) of the rice–fish farmers had changed from only rice farming to rice–fish farming. The main reason was that they had learned that rice–fish farming could increase their income. The fish yield had declined over the last 3 years for all rice–fish farmers, and most farmers felt that this was due to an overuse of pesticides. Farmers also perceived that the catch of wild fish had declined because of pesticides, the use of illegal fishing gear and loss of breeding habitat for aquatic animals, due to intensification of rice faming and the use of three crops per year.

### Agrochemicals and pest management strategies

The rice–fish farmers used less amounts of fertiliser than the rice farmers in both Cai Be and LSWR (Table 5). The farmers in LSWR used higher amount of fertilisers than the farmers in Cai Be (Table 5, *P* < 0.05).

**Table 5:** Average dose (kg ha^−1^ crop^−1^) of fertilisers among rice–fish (RF) and rice (R) farmers in Cai Be and rice (R) farmers in LSWR

	Type of farmer		
	Cai Be		LSWR
	RF	R	R
Nitrogen			
Mean	59.6^a^	65.9^a^	98.7^b^
SD	26.2	28.8	23.6
Phosphate			
Mean	42.1^a^	44.4^a^	83.5^b^
SD	35.4	23.4	18.7
Potassium			
Mean	64.2	86.6	73.0
SD	39.4	52.6	18.4
Total			
Mean	165.9^a^	196.9^a^	255.2^b^
SD	85.9	94.0	42.9

Means within the same row that do not share the same superscript letter are significantly different (*P* < 0.05)

*RF* farmers cultivating rice and farming fish, *R* farmers cultivating only rice

All farmers used pesticides as the main method to control pests. A total of 38 different pesticides and 35 different active ingredients (a.i.) were identified among the rice farmers in Cai Be. The rice–fish farmers in Cai be, used 36 different pesticides with 32 different active ingredients, which was slightly lower than the rice farmers. The highest number of pesticides was found in LSWR, with 40 different pesticides and 37 different active ingredients.

Fungicides and insecticides were the group of pesticides with the highest diversity. This could be due to problems with pesticide resistance in insects and fungal populations. The most problematic pest, mentioned by the farmers in Cai Be and LSWR, was the Brown planthopper (*Nilaparvata lugens*), which transmits a pathogenic virus and can cause significant crop losses. Brown planthopper was also a problem for the farmers in LSWR. The most commonly used insecticide, among all farmers was Chess 50 with the active ingredient pymetrozine.

Fungicides were commonly used by all farmers to control the rice blast disease caused by *Pyricularia oryzae*. The most commonly used fungicide was Anvil (a.i hexaconazole) in Cai Be and Amistar (a.i azoxystrobin and difenoconazole) in LSWR.

Herbicides and molluscicides were the group of pesticides with the lowest diversity. The most commonly used herbicide and molluscicide in Cai Be and LSWR were Sofit (a.i pretilachlor) and Toxbait (a.i metaldehyd), respectively.

All farmers said that they used pesticides that only killed target species. Rice–fish farmers were more cautious to use pesticides than the other farmers, because they had seen negative effects from pesticides on the fish. All farmers (except for two non-IPM farmers in Cai Be) knew about natural enemies to pests in their fields. The natural enemies that were mentioned were spiders, ants, bees, beetles, dragon flies, ladybugs and fish. All farmers knew that pesticides could kill these natural enemies, which in turn could lead to more problems with pests.

Most of the farmers in Cai Be, had learned how to use pesticides from governmental staff working at plant protection offices, which the rice farmers and rice–fish farmers on an average met 5 and 9.5 times per year, respectively. Most of the farmers in LSWR had learned how to use pesticides through personal experience and resellers. Five rice farmers met staff from the plant protection offices one to three times per year, while the other 15 rice farmers did not meet plant protection staff at all.

Many of the different pesticides were only applied by a small number of farmers. Rice–fish farmers used significantly lower number of different pesticides as compared to rice farmers, in LSWR (Table 6).

**Table 6:** Average number of different pesticides per crop used by rice–fish (RF) and rice (R) farmers in Cai be and rice (R) farmers in LSWR

	Type of farmer		
	Cai Be		LSWR
	RF	R	R
Insecticides			
Mean	1.5	2.0	1.5
SD	0.7	1.3	1.0
Herbicides			
Mean	1.0	1.0	1.3
SD	0.4	0.0	0.9
Fungicides			
Mean	2.0	1.9	2.6
SD	1.4	0.9	1.1
Molluscicides			
Mean	1.0	1.0	1.0
SD	0.2	0.3	0.7
Total			
Mean	5.4^a^	5.9^ab^	6.4^b^
SD	1.1	1.6	0.8

Means within the same row that do not share the same superscript letter are significantly different (*P* < 0.05)

*RF* farmers cultivating rice and farming fish, *R* farmers cultivating only rice

Rice–fish farmers also applied a significantly lower dose of insecticides compared to the farmers in LSWR. Their total use of pesticides also seemed to be lower compared to the other farmers, although this difference was not statistically significant (Table 7). The rice–fish farmers said that they had reduced their use of pesticides by around 40–50% during the last 3 years because of less pests and diseases. The non-IPM rice farmers in Cai Be had not changed their use of pesticides during the last 3 years. The farmers in LSWR had increased their use of pesticides by around 25%, because these farmers saw that pests had become more resistant to pesticides and their only solution to this was to increase the use of pesticides.

**Table 7:** Average dose (kg or l ha^−1^ spray^−1^) of active ingredient among rice–fish (RF) and rice (R) farmers in Cai Be and rice (R) farmers in LSWR

	Type of farmer		
	Cai Be		LSWR
	RF	R	R
Insecticides			
Mean	0.10^a^	0.55^ab^	0.36^b^
SD	0.13	1.50	0.52
Herbicides			
Mean	0.26	0.44	0.40
SD	0.48	0.25	0.46
Fungicides			
Mean	0.59	0.54	0.82
SD	0.67	0.43	0.57
Molluscicides			
Mean	0.43	0.63	0.72
SD	0.24	0.57	2.11
Total			
Mean	1.38	2.16	2.31
SD	0.97	1.82	2.62

Means within the same row that do not share the same superscript letter are significantly different (*P* < 0.05)

*RF* farmers cultivating rice and farming fish, *R* farmers cultivating only rice

**Fig. 2:**
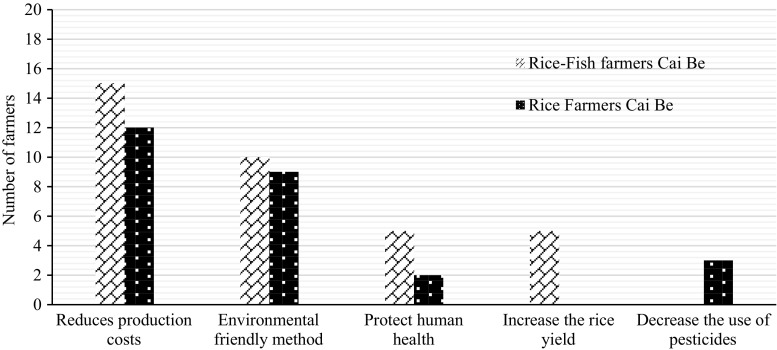
Main reasons for applying IPM among 20 rice–fish farmers (RF) and 14 rice farmers (R) in Cai Be

The farmers in Cai Be had learned about IPM from training courses. All rice–fish farmers had applied IPM for 3–12 years. The main reason for applying IPM was because it reduced the production costs (Fig. 2), and all of the farmers who applied IPM said that it had helped to increase their income. In LSWR, no farmer applied IPM because they thought it was difficult to combine with their rice farming practices.

### Health aspects

**Fig. 3:**
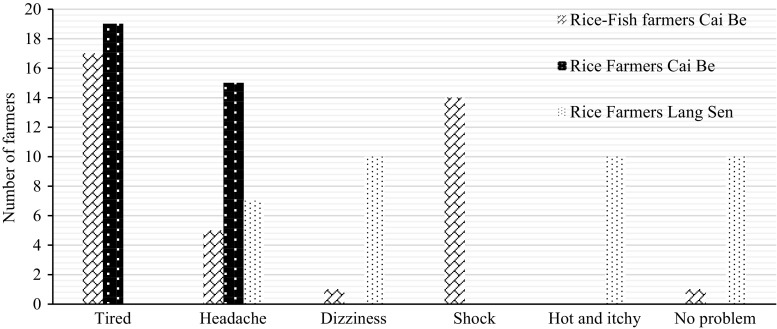
Health problems from pesticides among 20 rice–fish farmers (RF), 20 rice farmers (R) in Cai Be and 20 rice farmers (R) in LSWR

All farmers in Cai Be said that the pesticides had been a problem for their health. Insecticides were seen as the most harmful pesticide (Fig. 3). Almost all of the farmers used a mask as the only protection when spraying.

In LSWR, 10 rice farmers had experienced health problems related to pesticide use. Their symptoms were similar to the farmers in Cai Be such as dizziness and headache (Fig. 3). All the farmers in LSWR used mask and protective clothes.

### Financial aspects

All the rice–fish farmers said that rice–fish farming had increased their gross income by 10–30%. This was confirmed by the finding that the additional fish yield gave a 20 percent higher gross income for the rice–fish farmers as compared to farmers who cultivated only rice (*P* < 0.05, Table 8). Overall, the increased income from fish and decreased costs for fertilisers and pesticides resulted in a higher net income for the rice–fish farmers as compared to the other farmers, although this difference was not statistically significant. The selling price for rice varied to some extent between different rice varieties, which explains why the farmers in LSWR had a slightly higher income for rice despite their slightly lower rice yield compared to the rice farmers in Cai Be (Table 8).

**Table 8:** Cost and income (million VND ha^−1^ crop^−1^) among rice–fish farmers (RF) and rice farmers (R) in Cai Be and rice farmers in LSWR (R LSWR)

	Type of farmer		
	Cai Be		LSWR
	RF	R	R
Costs			
Rice seed			
Mean	1.50	1.56	1.41
SD	0.29	0.54	0.21
Fertilisers			
Mean	2.57^a^	3.52^a^	4.93^b^
SD	1.38	1.76	1.81
Pesticides			
Mean	1.71^a^	2.11^a^	3.00^b^
SD	0.89	1.32	1.18
Labour			
Mean	5.48	4.70	5.24
SD	1.23	1.60	1.08
Fish fingerlings^1^			
Mean	1.75	–	–
SD	0.68	–	–
Fish feed^1^			
Mean	4.93	–	–
SD	2.48	–	–
Chemicals^1^			
Mean	0.12	–	–
SD	0.03	–	–
Water			
Mean	–	–	0.66
SD	–	–	0.61
Total cost			
Mean	18.1^a^	11.9^b^	15.2^c^
SD	3.50	3.50	2.00
Gross income			
Rice crop			
Mean	30.8^a^	35.7^ab^	36.6^b^
SD	10.5	10.9	4.06
Cultured fish^1^			
Mean	12.3	–	–
SD	6.97		
Total gross income			
Mean	43.1^a^	35.7^b^	36.6^b^
SD	9.88	10.9	4.06
Net income			
Mean	25.0	23.8	21.3
SD	8.96	9.91	3.38

Means within the same row that do not share the same superscript letter are significantly different (*P* < 0.05)

*RF* farmers cultivating rice and farming fish, *R* farmers cultivating only rice

^1^The rice–fish farmers have one crop of fish per year. The costs and income for fish farming are divided by three, since the farmers had three crops of rice per year

Overall, the financial analysis shows that integrated rice–fish farming is a financially competitive alternative to rice monoculture, and that it uses less pesticides and fertilisers, which is likely to have positive effects on the environment and the farmers’ health, with additional positive long-term economic effects.

### Farmers’ perception of ecosystem services status and trends

**Fig. 4:**
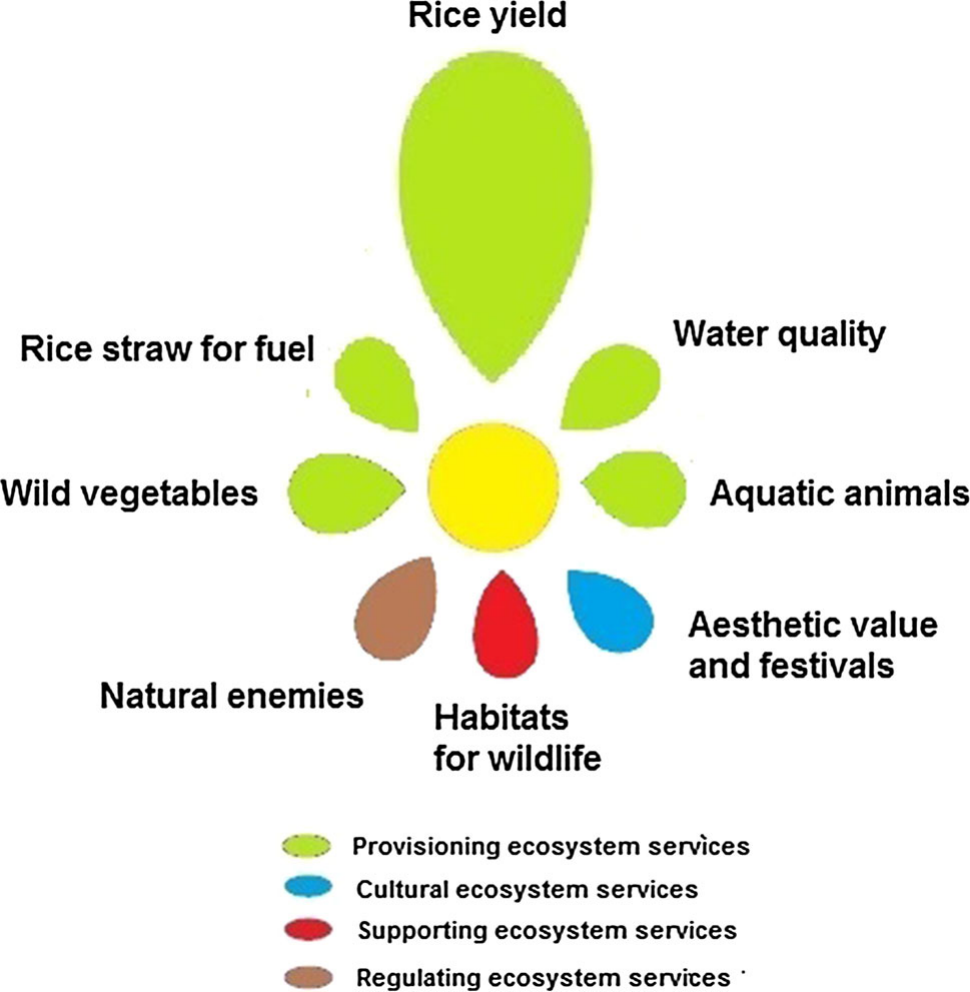
The overall perception of the farmers was that an increased production of rice has led to a general decrease of other key ecosystem services. The figure is a qualitative illustration of the farmers’ perception of the status of key ecosystem services provided by the rice fields and related wetlands in the Mekong Delta Adapted from Gordon et al. 2010

As shown in the previous financial analysis, rice has a large and direct impact on the farmers’ income and all farmers said that the rice yield was the most important service gained from the rice field ecosystem (Table 9). Still, the farmers also identified a number of other ecosystem services of importance to their livelihoods and wellbeing (Fig. 4).

**Table 9:** Ranking of the importance of ecosystem services by the different farmer groups

Rank	Rice farmers in Cai Be	Rice–fish farmers in Cai Be	Rice farmers in LSWR
1	Rice yield	Rice yield	Rice yield
2	Water quality	Water quality	Aquatic animals
3	Habitats for wildlife	Habitats for wildlife	Water quality
4	Aquatic animals	Aquatic animals	Wild vegetables
5	Wild vegetables	Wild vegetables	Habitats for wildlife
6	Natural enemies	Natural enemies	Rice straw for fuel
7	Aesthetic value and festivals	Rice straw for fuel	Natural enemies
8	Rice straw for fuel	Aesthetic value and festivals	Aesthetic value and festivals

Provisioning services included, in addition to rice, clean water, aquatic animals, wild vegetables and fuels. Among supporting services, habitats for wildlife and soil structure, were most commonly mentioned. Regulating services such as pollinators and natural enemies to control pests and diseases, were identified as important. Cultural services such as aesthetic values and festivals, were not so commonly mentioned by the farmers, but still seen as important (Fig. 4; Table 9). Overall, provisioning services seemed to be easiest for the farmers to understand and directly relate to.

### Trends in the abundance of key ecosystem services

The farmers felt that during the last 15 years, there had been an increased production of rice but a decrease in many of the other ecosystem services (Fig. 4; Table 10). Contrary to the farmers in Cai Be, 35% of the farmers in LSWR had experienced an improved water quality during the last 15 years.

**Table 10:** Farmer’s perception of changes in key ecosystem services during the last 15 years

Ecosystem service	Rice farming Cai Be	Rice–fish farming Cai Be	Rice farming LSWR
Rice yield	↑20^a^	↑20	↑20
Aquatic animals	↓20	↓20	↓20
Wild vegetables	↓20	↓20	↑2↓18
Water quality	↓20	↓20	↑7↓13
Rice straw for fuel	↑1↓19	↓20	↑1↓19
Habitats for wildlife	↑1↓19	↓20	↑3↓17
Aesthetic value and festivals	↑1↓19	→1↓19	↓20
Natural enemies	↑2↓18	↑1↓19	↑2↓18

The arrows indicate increase (↑), decreased (↓) or no change (→)

^a^Number of farmers (total 20)

The future trend, for the coming 15 years, was perceived mostly as a continued increased production of rice and a continued decrease of the other key ecosystem services (Table 11). However, 19 farmers from the three different groups thought that natural enemies would increase.

**Table 11:** Farmer’s perception of change in key ecosystem services for the next coming 15 years

Ecosystem service	Rice farming Cai Be	Rice–fish farming Cai Be	Rice farming LSWR
Rice yield	↑14→5↓1	↑18→2	↑17→3
Aquatic animals	↑1↓19	↑1→1↓18	→2↓18
Wild vegetables	↑1→1↓18	14→6↓	↑2^a^↓17
Water quality	↑1↓19	↑1↓19	↑3→4↓13
Rice straw for fuel	↑2→3↓15	↑4↓16	↑3→1↓16
Habitats for wildlife	↑3↓17	↑1→2↓17	↑3→1↓16
Aesthetic value and festivals	↑1→4↓15	↑1→3↓16	↑2↓18
Natural enemies	↑8↓12	↑4↓16	↑7↓13

^a^Significantly different (*P* < 0.05)

The arrows indicate increase (↑), decrease (↓) or no change (→)

### Rice yield

**Fig. 5:**
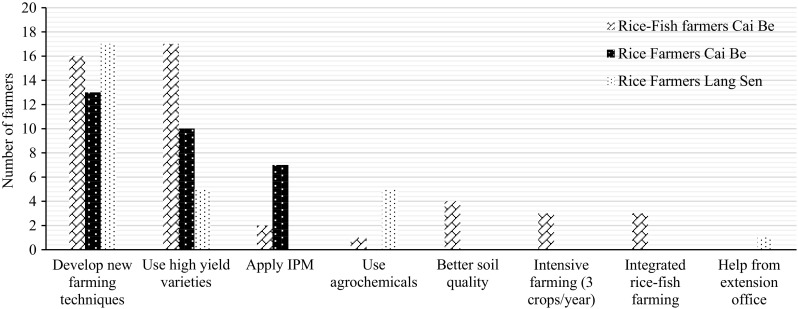
Identified reasons for increased rice yields according to the farmers

All farmers said that their rice yields had increased during the last 15 years (Table 10). The majority of farmers felt that the rice yields would continue to increase. However, some farmers, especially intensive rice farmers in Cai Be, thought that the rice yield had peaked and would not be possible to increase in the coming 15 years (Table 11). The most common reasons for the increased rice production were the introduction of high-yielding rice varieties and new farming techniques, including IPM (Fig. 5).

None of the rice farmers in LSWR, but 13 rice–fish farmers and 9 rice farmers in Cai Be, thought that pesticides could have a negative effect on the rice yield. These farmers had experienced that the use of pesticides could result in resistant pests, increased disease problems and decreased populations of natural enemies to the rice pests.

In LSWR, the farmers said that they would like to have three crops per year because that would increase their income, but it was difficult for a single farmer to switch to three crops if not all neighbouring farmers did the same.

### Aquatic animals

**Fig. 6:**
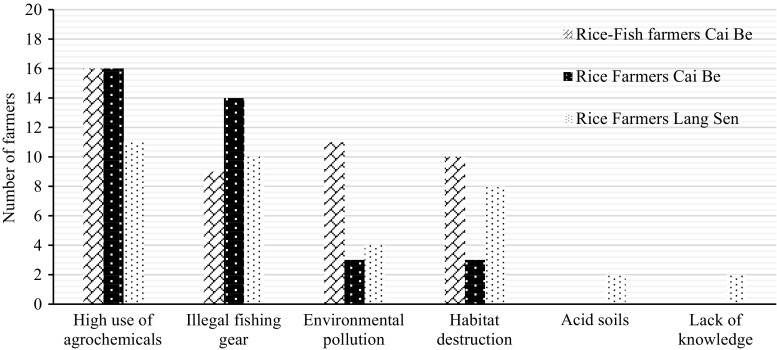
Identified reasons for the decrease of aquatic animals according to the farmers

All farmers said that the number of aquatic animals found in the rice fields and related wetlands had decreased during the last 15 years, and the majority said that this trend would continue (Table 10, 11). All farmers felt that the high use of pesticides was the biggest problem (Fig. 6).

The rice–fish farmers also mentioned that intensive farming, with three crops per year, was a reason for the increased loss of aquatic animals (Fig. 6). Having one or two crops per year was seen as beneficial for aquatic animals, since it gave them more time and space to breed and feed. One farmer in Cai Be said that he used to harvest around 100 kg of wild fish per year, but today, he only caught around 40 kg due to more intensive rice farming. Some older villagers in LSWR said that fish were not as plentiful as they used to be, and that the size of the fish was much smaller than before.

All farmers felt that a decreased use of agrochemicals would help to halt the loss of aquatic animals. Rice–fish farmers said that integrated rice–fish system could contribute to an increased abundance of aquatic animals. Seven of the farmers in LSWR emphasised the need for more education to improve the situation.

### Wild vegetables

**Fig. 7:**
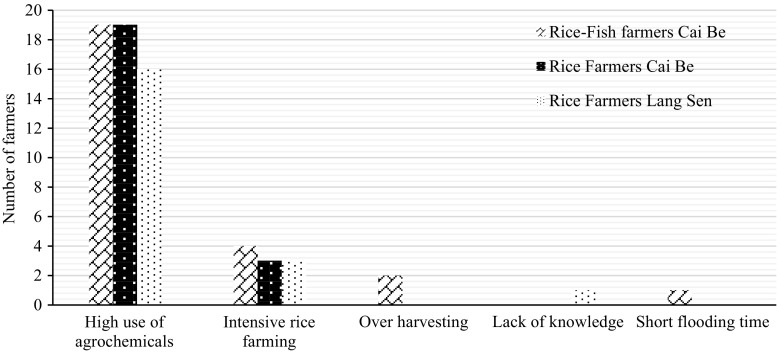
Identified reasons for the decrease of wild vegetables according to the farmers

The majority of farmers thought that the abundance of wild vegetables had decreased (Table 10), mainly because of an overuse of agrochemicals (Fig. 7).

The most common solution proposed to stop the decrease in wild vegetables was to minimise the use of agrochemicals, and especially herbicides. The rice farmers in Cai Be also saw IPM as a way to improve the conditions for wild vegetables and plants. Rice–fish farmers said that intensive farming and the lost connectivity between rice fields and surrounding areas had contributed to the loss. To improve the situation, they proposed to integrate wild vegetables with rice, both in the canals and on the dikes. Wild vegetables could provide food for fish and also be used for their own consumption and for sale. The idea of having a rotation between rice and vegetables was also mentioned by several of the rice farmers in Cai Be, which could be a sustainable alternative of having three crops of rice per year.

### Water quality

The majority of farmers in Cai Be felt that the water quality had decreased and was going to get worse in the future (Tables 10, 11). None of the farmers used the water for household consumption anymore. They stopped using it 10 years ago, since the quality had decreased a lot, and people experienced negative health aspects like rashes from exposure to the water. None of the farmers in Cai Be believed that the water quality could get good enough to use it for household consumption again. They believed that the change had gone too far to reverse. The farmers were concerned about how upstream activities impacted on the water quality. The rice–fish farmers said that they closed their water intake when neighbouring rice famers released water from their rice fields, to stop the polluted water from entering into the fields, since they had experienced negative effect on their fish. Some of the farmers also had rice fields without fish as a buffer zone between their rice–fish fields and the surrounding rice fields, to avoid getting pesticides into their rice–fish fields.

**Fig. 8:**
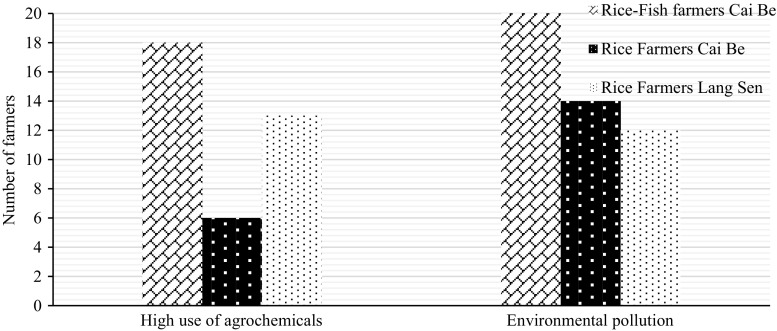
Identified reasons for the decrease of water quality according to the farmers

All farmers felt that the use of agrochemicals and the environmental pollution from farms, households and industries, were the main reasons for the decreased water quality (Fig. 8). Seven of the farmers in LSWR, however, said that the quality of the water had improved due to measures taken against acid sulphate soils (flushing out iron sulphide). Many farmers in LSWR also said that they still used the water for household consumption and drinking. However, many of them said that the water quality had decreased, due to chemicals from rice fields and they were concerned that an overuse of pesticides and wastewater would reduce the water quality in the future.

A reduced use of chemicals and better wastewater treatment systems were seen as possible solutions to improve the water quality by all farmers. Some farmer said that these measures needed to be supported by improved education.

### Rice straw

**Fig. 9:**
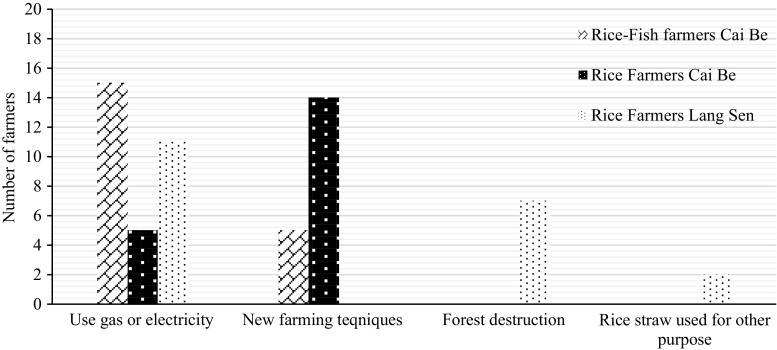
Identified reasons for the decrease of rice straws for fuel according to the farmers

Most farmers felt that the use of rice straw for fuel was increasingly being replaced by gas or electricity (Fig. 9). Many farmers mentioned that rice straws could be beneficial in other ways, such as substrate for mushrooms or as food for animals.

### Natural enemies

**Fig. 10:**
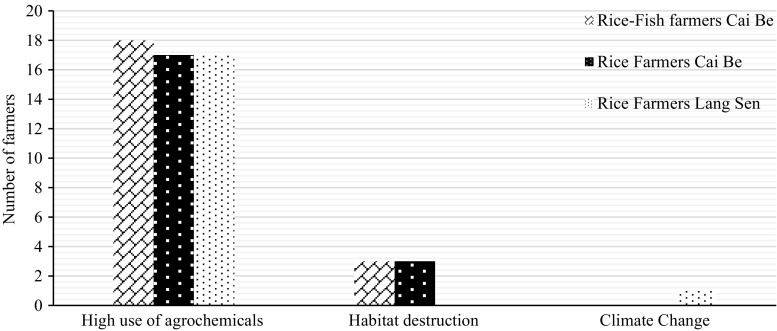
Identified reasons for the decrease of natural enemies according to the farmers

Most farmers felt that there had been a decline in natural enemies during the last 15 years (Table 10), mainly because of a high use of agrochemicals and habitat destruction (Fig. 10). Proposed solutions to the decline in natural enemies included the use of less toxic and lower amounts of pesticides. Many of the farmers in Cai Be also mentioned that IPM builds on protecting natural enemies to control rice pest, and, therefore, could help to both reduce the use of pesticides and increase the number of natural enemies. Some farmers felt that the number of natural enemies had increased and would continue to do so in the future, because people increasingly knew about the benefits from natural enemies and, therefore, were willing to protect them.

### Habitat for wildlife

**Fig. 11:**
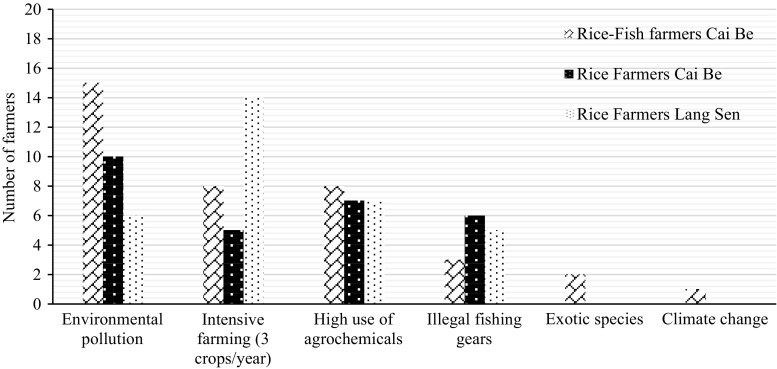
Identified reasons for the decrease of habitats for wildlife according to the farmers

The majority of farmers said that habitats for wildlife had decreased compared to 15 years ago (Table 10). Environmental pollution, intensive farming, high use of agrochemicals and illegal fishing gears were mentioned as the most common reasons for the decline (Fig. 11). Farmers in Cai Be said that the low water levels kept for the rice created problems for aquatic organisms and that the use of machines, instead of harvest by hand, had limited the breeding time for many animals.

Some farmers in Cai Be expected that the habitat situation would improve in the future (Table 11) because of the use of IPM and because people had started to recognise the importance of ecosystem services for a sustained production of rice. In LSWR, three famers said that the habitat status was good and would continue to be so, because they only had two crops of rice per year, as the fields were flooded during the time for the third crop. They felt that this and the closeness to the reserve area safeguarded a good habitat for wildlife.

All farmers felt that a decreased use of agrochemicals and increased protection of habitats would improve the status of habits for wildlife. Farmers from Cai Be also mentioned that stronger laws against illegal fishing, having two crops instead of three crops, treating wastewater, integrating rice with other crops and education, would help to improve the situation.

### Aesthetic values and festivals

**Fig. 12:**
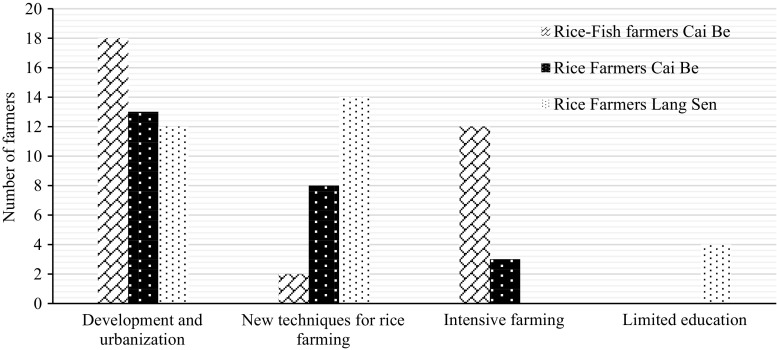
Identified reasons for the decrease of cultural services according to the farmers

Cultural services were the most difficult services for the farmers to understand, although most farmers could relate to aesthetic values and festivals after some discussions and explanations. Some farmers also talked about the importance of rice fields for generating employment. The majority of farmers experienced a decline in cultural services due to intensive farming systems, use of machines and urbanization (Table 10; Fig. 12). All farmers felt that younger people did not see the countryside in the same way as older people. They did not appreciate the aesthetic value and the life of being a rice farmer, but preferred to move to the cities to work (Fig. 12). This was believed to be the future trend (Table 11). However, some farmers believed that cultural ecosystem services would increase in the future, because of better income and more festivals.

Several of the farmers did not know how to enhance the status of cultural services, but a few mentioned that education could help to increase the awareness of cultural services. Some of the rice–fish farmers and farmers in LSWR mentioned ecotourism as a way to enhance cultural services, and the need to preserve old farming methods.

### Preferences to future farming systems

**Fig. 13:**
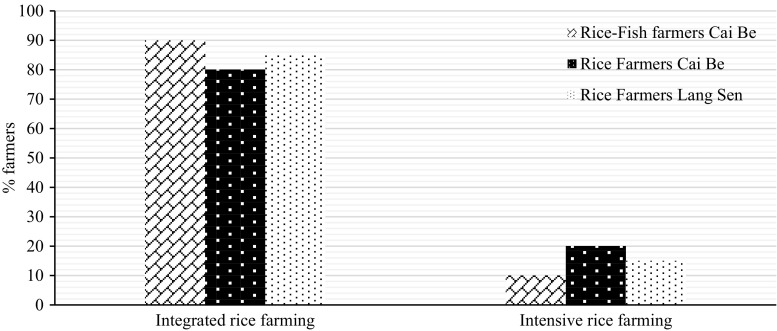
Percentage of rice farmers and rice–fish farmers in Cai Be and LSWR, who were in favour of intensive rice monocropping, with a high rice yield but lower quality of other ecosystem services, or more extensive integrated farming systems, with lower rice yield but higher quality of other ecosystem services

In Cai Be and Lang Sen, 20 and 15% of the rice farmers, respectively, preferred intensive rice farming with the main aim to produce high yields of rice, whereas 80 and 85% of the farmers, respectively, preferred rice farming systems that would enhance or preserve multiple ecosystem services at the expense of somewhat decreased rice yields (Fig. 13).

However, if the rice yield would become less than 6 tons per crop, they might re-consider their choice. In Cai Be, the farmers felt that three crops of rice per year was necessary to get enough income, even though they were aware that this could have a negative effect on the yield, since the rice field did not have time to recover between the crops. They also knew that these intensive farming strategies could cause increased problems with diseases and other pests. However, they could not see any option due to the high competition and the low market price for rice.

Only 10% of the rice–fish farmers in Cai Be preferred a system with a high yield of rice, and 90% preferred rice farming systems with lower rice yield, but that would help to keep all ecosystem services in a good quality (Fig. 13).

The rice–fish farmers said that an integrated system could provide both many ecosystem services and an acceptable rice yield, but that this was hard to establish since neighbouring farmers used a lot of pesticides that influenced negatively on their rice–fish fields. They also said that they could not accept their rice yield to decrease too much since it was their main income. They were positive about the income from fish, and wanted to continue with integrated rice–fish farming, because it diversified their income opportunities and provided benefits to the environment.

## Discussion and conclusion

Being one of the world’s largest tropical wetland areas, the Mekong Delta provides suitable conditions for rice farming and is the most important rice production region in Vietnam, contributing to the national food security and income (Chapman and Darby 2016). Increased rice yields have provided export earnings and help reduce poverty. However, as shown by this study, this has come at the expense of other ecosystem services of importance to people’s livelihoods and wellbeing (Berg et al. 2012; Sebesvari et al. 2012).

Many farmers felt that the high use of pesticides and three crops per year had led to a decline in most of the identified key ecosystem services, and the economic rationale of having three crops per year is increasingly being questioned (Garschagen et al. 2012). The rice from the third crop is often of low quality and any extra income is easily offset by the need to buy more pesticides and fertilisers (Chapman and Darby 2016; UNEP 2005). Earlier studies show that farmers with two crops per year have a higher income per crop than farmers with three crops per year, indicating decreased production efficiency with increased production intensity (Berg 2002). Thus, the rice production in intensively cultivated areas, such as Cai Be, may have reached an upper limit where increased yields can only be achieved through increased inputs of fertilisers and pesticides, with decreasing net incomes for the farmer and increasing negative impacts on the environment and peoples’ health (Berg et al. 2012; Chapman and Darby 2016).

Still, intensive farming with three crops per year has been encouraged by governmental policies (Chapman and Darby 2016; Garschagen et al. 2012), and some farmers felt that although the financial benefits from a third crop may be minor, it still provided some rice, which is better than nothing. The option to not have a third crop may be difficult to accept for farmers with a small income, who also have been encouraged by the government to have three crops as a means to increase the rice production in the Delta over the years (Chapman and Darby 2016).

All farmers said that the number of aquatic animals found in rice fields and related wetlands had decreased during the last 15 years, and the majority said that this trend would continue. Most farmers also thought that there had been a decrease in the abundance of wild vegetables. High use of agrochemicals and intensive rice farming were often mentioned as the main reasons for the decline in aquatic animals and vegetables. This was also seen as main causes for the decline in water quality, both in the past and in the future.

This has implications for the livelihoods and wellbeing of many different stakeholders and for the overall food production in the Delta. Especially, poor people depend on wild aquatic resource and are vulnerable to a decreased quality of the Delta’s water resources (MRC 2010). The Mekong Delta is one of the poorest and most densely populated areas in Vietnam (Renaud and Kunezer 2012), and care must be taken to safeguard these peoples’ livelihoods and wellbeing under future development scenarios.

In Cai Be, none of the farmers used river water for household consumption anymore, and the majority relied on water from water treatment plants. The rice–fish farmers also said that the low water quality had impacted negatively on their fish. Pesticides used on rice fields spread easily to other areas, and some farmers did not start with rice–fish farming because of the high use of pesticides on neighbouring rice fields. This clearly illustrates how the services of clean water (and fish) have been compromised for increased production of rice, and where water is increasingly seen as a “dis”-service impacting on peoples’ health and the environment. Such losses of ecosystem services, not only have direct impact on people’s wellbeing, but also restrict future options for alternative and diversified livelihoods, which may be critical for local people’s ability to adapt to changes following from upstream dams and climate change (MRC 2010; Nguyen and Woodroffe 2015; Smajgl et al. 2015; Tessler et al. 2016; Vogt et al. 2016).

This lesson must be taken seriously as the ecosystem services of the Mekong Delta support many different stakeholders, with a high diversity of different activities and livelihoods. It must be made more clear how these activities depend and impact on the Delta’s ecosystem services, to avoid unwanted trade-offs, where long-term benefits from multiple ecosystem services are lost for short-term financial gains from single crops.

For example, the aquaculture industry in the Mekong Delta, which is expanding quickly, is highly dependent on clean water for an efficient and high-quality production of fish, and there is an urgent need to balance this against the intensification and increased use of agrochemicals in rice farming and other agriculture sectors (De Silva and Phuong 2011).

Thus, there is a need to rethink the production of food so that future strategies limit the impact on, or preferably enhance, different ecosystem services, to safeguard the environment and the long-term production of food in the Delta (Johnston et al. 2010; Smajgl et al. 2015, Zheng et al. 2016). Future strategies should aim to avoid an overuse of agrochemicals and improve the production efficiency through increased recycling of nutrients and matter.

As indicated in this study, and by several other studies, integrated rice–fish farming, for example, provides a competitive alternative to intensive rice monoculture with several environmental advantages (Berg 2002; Berg and Tam 2012; Devendra and Thomas 2002; Xie et al. 2011; Zheng et al. 2016). Rice–fish farmers had a more selective use of pesticides and used less numbers of different pesticides (Table 6) and lower doses of pesticides (Table 7), as compared to intensive rice farmers.

The rice–fish farmers said that they had reduced their use of pesticides by around 40–50% during the last 3 years. This could be partly because they know that the fish act as natural enemy to the rice pests and that the pesticides impact on the fish productivity (c.f. Zheng et al. 2016). These farmers only applied pesticides when they saw pests in the field, and not as a prevention method. Rice–fish farmers also used less fertiliser than rice farmers (Table 5), partly because the fish can help to enhance the nutritional status of the rice field environment (Tsuruta et al. 2011; Xie et al. 2011, Zheng et al. 2016). These evidences of benefits from ecosystem services probably explain why rice–fish farmers were in the strongest support of future scenarios dominated by integrated farming systems. The supplementary income from fish also seemed to make them more willing to accept a decreased rice yield, as this tentatively could be compensated by an increased fish yield. Thus, it seems that integrated systems help to create a number of positive feedbacks between the rice field environment and the farmers’ income, helping the farmer to recognise the benefit from ecosystem services, and encouraging the farmer to adopt new and more sustainable farming strategies. An increased reliance on ecosystem services for pest control and fertilisation helped to reduce the production costs and to increase the net income for these farmers (c.f. Zheng et al. 2016).

Many of the rice farmers in Cai Be and LSWR, on the other hand, felt that there has been a decline in natural enemies during the last 15 years (Table 10) and complained about resistant pests, which they tried to counteract through an increased use of pesticides. These are common effects from continued high use of pesticides (Spangenberg et al. 2015; Wilby and Thomas 2002), and illustrate how easily the choice of farming strategies could disrupt rather than enhance ecosystem services, such as natural enemies to pests (Luo et al. 2014). Although many rice farmers were aware of some of the negative environmental consequences, they could not see any option to intensive rice farming, due to the high competition and the low market price for rice. In this case, the farmers have been locked into negative feedbacks between the environment and the farming system, where declining ecosystem services, such as natural enemies to rice pests and soil fertility, needs to be compensated by increasing inputs of fertilisers and pesticides (Chapman and Darby 2016). Understanding and balancing trade-offs in ecosystem services is complex and need support in terms of education and awareness building, which was highlighted several times by the farmers. For example, many farmers burn their rice straws to recycle nutrients to the soil, but then also destroy valuable habitats for natural enemies to rice pests (Luo et al. 2014). Also, governmental policies to increase the rice production should encourage farmers to adopt farming strategies with an increased production efficiency, rather than adopting three crops per year, which has been an important strategy in the previous years and, probably, still influences farmers’ behaviours (Chapman and Darby 2016).

All rice–fish farmers said that rice–fish farming had increased their income, which may be the main argument for the farmer to change from rice farming to rice–fish farming. However, there are also other less obvious benefits, which, in the long run, may be important arguments for the farmer to continue with rice–fish farming. Some farmers mentioned, for example, that a decreased use of pesticides would help to improve the water quality and the farmers’ health.

Almost all of the farmers in both the study areas complained about health problems related to the use of pesticides. Insecticides were commonly mentioned as the most harmful pesticides, which is similar to earlier findings (Berg 2001, 2007; Berg and Tam 2012; Dasgupta et al. 2007). The most common health problems described by the farmers were fatigue and headache. Many farmers also felt tired after spraying, which could be an early symptom of nervous-system effects from exposure to organophosphates and carbamates, which are commonly used pesticides in the Mekong Delta (Dasgupta et al. 2007; Tam et al. 2015). Even though most farmers experienced health effects from pesticides, they saw no alternative to pesticides, since it was considered the most efficient measure to prevent pest outbreaks. This was also a common statement found in a survey among farmers in Vietnam by Toan (2011).

Another benefit with rice–fish farming, mentioned by several farmers, was that the more diversified and less intensive production improved the status of habitats for wild species and the overall biodiversity of the rice field ecosystem (cf. Luo et al. 2014). In LSWR, farmers felt that the habitat status was good because they only had two crops. Longer periods of flooding and the closeness to the reserve area also safeguarded a good habitat for aquatic organisms (cf. Luo et al. 2014). The lack of this kind of “connectivity” between different habitats was highlighted as a problem in Cai Be. The farmers said that three crops, with higher dykes and shorter and more controlled flooding, had led to a decreased connectivity between the rice fields and the surrounding areas, contributing to the loss of wild aquatic species. This confirms the review by Luo et al. (2014), who found that the intensification of rice farming in China, with changed irrigation systems had impacted on the habitat of the rice fields, and reduced the amount and types of species. Measures to keep or increase the connectivity between water and the rice fields are important to increase the diversity of animals and plants, which provides the basis for the systems’ resilience and provision of multiple ecosystem services (Luo et al. 2014). Many rice farmers in Cai Be felt that the rotation between rice and vegetables could also diversify the production, and provide a sustainable alternative of having three crops of rice per year. Many rice–fish farmers proposed to integrate rice with other crops or wild plants, which would provide food and shelter to the fish, and could be used for their own consumption or sale. They also felt that one or two crops per year would benefit aquatic animals since it gives more time for them to breed and more space for them to thrive.

Increased connectivity and diversity are key factors for designing systems that are resilient to change (Tessler et al. 2016; Vogt et al. 2016; Walker and Salt 2006), and should constitute important components when designing future agriculture systems in the Mekong Delta (Kunezer and Renaud 2012). The social ecological systems of the Delta have been shaped by the annual fluctuation of the Mekong River. These systems are likely to be exposed to even more variable conditions in the future, following from climate change and upstream dams (Smajgl et al. 2015; MRC 2010), and future strategies should build on these systems’ intrinsic abilities to adapt and “live with change”, to develop systems with high general resilience (Walker and Salt 2006). However, it is important to act on emerging and remaining opportunities, as a recent study by Smajgl et al. (2015) indicates that the adaptive capacity of central provinces in the Mekong Delta has become very low. Proposed adaptation strategies to climate change and upstream dams include changing from two or three crops of rice to a mixed regime of rice and aquaculture (Smajgl et al. 2015). As indicated by our results, this would build on and take advantage of existing knowledge of these systems in the Mekong Delta, and would probably not only help local communities to adapt to future changes, but also provide options for diversified and sustainable food production systems, and improve farmer’s income (Smajgl et al. 2015).

An overall strategy for enhanced adaptability and resilience is also to safeguard the status and diversity of ecosystem services (Walker and Salt 2006). This requires an improved awareness of the multiple benefits delivered by ecosystem services among different stakeholders, and as mentioned repeatedly by the farmers, education and training are keys to move society toward sustainable rice farming strategies (Luo et al. 2014). It is vital that different stakeholders, including farmers and governmental officers, recognise the significance of the ecosystem services provided by rice fields and associated wetlands, and also understand the pathways to protect and restore rice field biodiversity and the multiple ecosystem services that they provide (Luo et al. 2014).

Thus, the benefits derived from ecosystem services must be increasingly recognised and considered in the development of future agriculture systems of the Mekong Delta. Strategies should be directed toward methods that make use of the natural environment without severely or irreversibly degrading it. Our study indicates that this would not only make financial sense to the individual farmer, but also benefit the whole region in the long run, through an improved status of the environment and peoples’ health.
